# Protocol of supra-visceral aortic ischemic preconditioning for open surgical repair of thoracoabdominal aortic aneurysm: The EPICATA study (Evaluation of the Efficacy of Ischemic PreConditioning on morbidity and mortality in open ThoracoAbdominal Aortic surgery)

**DOI:** 10.1186/s12893-020-00851-3

**Published:** 2020-08-27

**Authors:** Mickael Palmier, Mickael Bubenheim, Laurent Chiche, Xavier Chaufour, Fabien Koskas, Elie Fadel, Pierre Edouard Magnan, Eric Ducasse, Nabil Chakfe, Eric Steinmetz, Marie Melody Dusseaux, Jean Baptiste Ricco, Didier Plissonnier

**Affiliations:** 1 Department of vascular surgery and Inserm U1096, Rouen University Hospital, 1 rue de Germont, 76031 Rouen Cedex, France; 2 Department of Clinical research and Innovation, Rouen University Hospital, Rouen, France; 3 Department of vascular surgery, Pitié-Salpétrière University Hospital, Paris, France; 4 Department of vascular surgery, Rangueil University Hospital, Toulouse, France; 5 Centre Chirurgical Marie Lannelongue, Le Plessis Robinson, France; 6 Department of vascular surgery, La Timone University Hospital, Marseille, France; 7 Department of vascular surgery, Pellegrin University Hospital, Bordeaux, France; 8 Department of vascular surgery, Nouvel Hopital Civil, University of Strasbourg, Strasbourg, France; 9 Department of vascular surgery, Dijon University Hospital, Dijon, France; 10 Department of anesthesia, Rouen University Hospital, Rouen, France; 11 Department of Clinical Research and Innovation, University Hospital, Poitiers, France

**Keywords:** Thoracoabdominal aortic aneurysm, Preconditioning, Pulmonary and renal morbidity

## Abstract

**Background:**

Open surgical repair (OSR) for thoracoabdominal aortic aneurysms (TAA) is associated with a high pulmonary and renal morbidity rate. Ischemic preconditioning (IPC) is a mechanism of protection against the deleterious effects of ischemia-reperfusion. To our knowledge IPC has never been tested during OSR for TAA.

**Methods:**

The primary objective of the study is to evaluate the efficacy of IPC during OSR for TAA with respect to acute kidney injury (AKI) according to KDIGO and pneumonia/prolonged ventilation-time during the first 8 postoperative days. The secondary objectives are to compare both arms with respect to cardiac complications within 48 h, renal and pulmonary complications within 21 days and mortality at 60 days.

To assess the efficacy of IPC with respect to pulmonary and renal morbidity, a cox model for competing risks will be used. Assuming that the event occurs among 36% of the patients when no IPC is performed, the allocation of 55 patients to each arm should allow detecting a hazard ratio of at least 2.75 with a power of 80% when admitting 5% for an error of first kind. This means that 110 patients, enrolled in this multicenter study, may be randomised within 36 months of the first randomization.

Randomization will be performed to allocate patients either to surgery with preconditioning before aortic cross clamping (Arm 1) or to surgery without preconditioning before aortic cross clamping (Arm 2).

Randomization takes place during the intervention after intravenous injection of heparin, or after the start of femoral assistance. The procedure for IPC will be a supra-visceral thoracic aortic cross clamping for 5 min followed by an unclamping period of 5 min. This procedure will be repeated twice before starting thoracic aortic cross clamping needed to perform surgery.

**Conclusions:**

Our hypothesis is that ischemic preconditioning could reduce clinical morbidity and the incidence of lung damage associated with supra-visceral aortic clamping.

**Trial registration:**

EPICATAStudy registered in ClinicalTrial.gov / number: NCT03718312 on Oct.24.2018 URL number

## Background

Mortality rate after OSR for TAA is estimated between 4 and 15% [1, 2]. Pulmonary and renal complications can reach up to 50% and more [3, 4]. Compared to OSR for infrarenal abdominal aortic aneurysm (AAA), TAA requires repair of the visceral arteries with a supravisceral aortic cross-clamping often greater than 30 min. Following OSR for TAA, Serious lung damage reached 60% in large series [3, 5] and 40 to 50% in recent publications [6]. The hypothesis of a causal link between visceral ischemia and the occurrence of organ failure and particularly lung damage is the main focus of this study. Mesenteric ischemia reperfusion caused by aortic cross clamping above the celiac trunk and the superior mesenteric artery is involved in the onset of a systemic inflammatory response (SIRS) leading to multiple organ failure (MOF) [7–10]. Interestingly, distal perfusion of the aorta by a shunt reduces the onset of paraplegia, but increases the production of cytokines and complications associated with the inflammatory response [11, 12].

The main actors of the inflammatory response to ischemia-reperfusion are free radicals’ oxygen, polymorphonuclear neutrophil cells (PMN) and cytokines, all at the origin of the deleterious complications [13–15]. In particular, activity of neutrophils could be a key factor in remote lung injury [16, 17].

Beside remote lung injury, renal damage can be mainly attributed to direct effect of cross aortic clamping during TAA. Severe acute renal failure reaches 25% with dialysis required in 17% of patients [6] with an increased risk of death [18]. Wynn et al. reported that renal failure as defined in the RIFLE classification [19] was not associated with increased mortality [20]. According to Tshomba et al. [21] acute renal failure is often temporary. But even temporary, it is possible that acute renal failure observed in this context may later favour the onset of chronic renal failure [22].

IPC applied to OSR for AAA was reported to reduce myocardial and renal damage [23]. But this result has been debated and other studies have reported negative results with IPC during AAA OSR [24] or peripheral vascular surgery [25]. IPC has been shown to improve lung function measured by blood gases and mesenteric aggression measured by lactate blood level [26]. Finally, a recent meta-analysis of 9 randomized trials, focused on the effects of remote IPC in patients operated for AAA, through an open or an endovascular approach, failed to report any beneficial effect on mortality, myocardial ischemia and renal impairment [27]. But these series were carried out during OSR of infrarenal AAA where the visceral arteries were not involved in aortic reconstruction and by that ischemia reperfusion damage [24, 25]. In these conditions, the protective effects of IPC appear difficult to evaluate [27–29]. In the same way, the CRIPES studying EVAR, which required no aortic clamping, did not show any protective effect of IPC on myocardial function [30]. On the contrary, a recent meta-analysis showed that IPC performed by clamping the thoracic aorta while performing coronary bypass, reduced the duration of postoperative artificial ventilation and myocardial ischemia [31].

### Rationale of the study

Our hypothesis is the existence of a causal relationship between visceral ischemia and remote organ failure. Visceral aortic cross clamping causes mesenteric and renal cells ischemia reperfusion injury leading to an intense and systemic inflammatory activity which could affect remote organs.

IPC may be a useful tool for endogenous protection against the deleterious effects of ischemia-reperfusion has been already demonstrated in myocardial ischemia [32], liver and kidney ischemia [33], but IPC for direct OSR of TAA requiring supravisceral aortic cross clamping has never been tested.

## Methods/design

### Trial objective

The primary objective of the study is to evaluate the efficacy of IPC applied directly to the supravisceral aorta during OSR for TAA with respect to acute kidney injury (AKI) according to KDIGO and pneumonia/prolonged ventilation-time during the 8-day postoperative period.

The secondary objectives are to compare both arms with respect to cardiac complications within 48 h, renal and pulmonary complications within 21 days and mortality at 60 days.

### Trial design

**Fig. 1:**
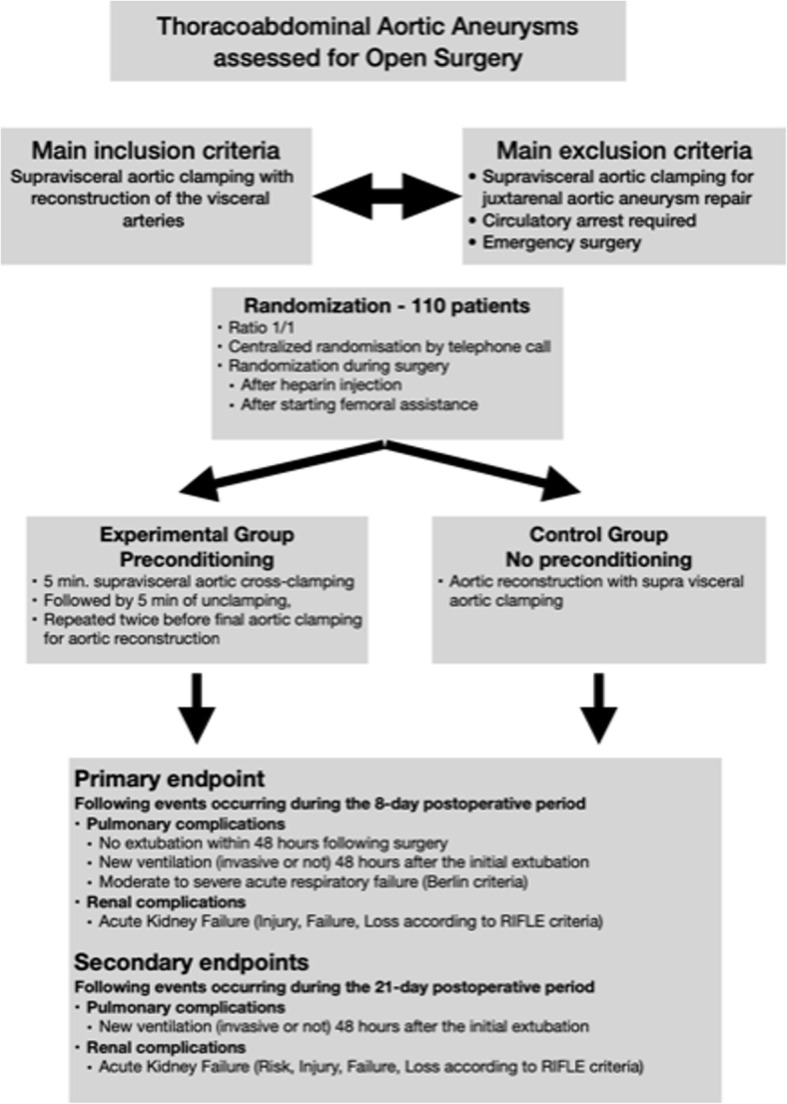
Study protocol flow chart

EPICATA is a randomized, open, multicentric national French study, the essential aspects of which have been summarized on a flowchart (Fig. 1).

### Primay endpoint

The primary endpoint is the occurrence of a first pulmonary or renal complication during the 8-day postoperative period.

Pulmonary complications are defined as:

- The necessity of prolonged artificial ventilation, i.e. no extubation before the 48-h following the surgery
- The need for a new ventilation (invasive or noninvasive) within 48-h following initial extubation
- The occurrence of a moderate or severe acute respiratory failure, according to the Berlin definition [34].

Renal complication is defined as the occurrence of an acute kidney failure according to the RIFLE classification [19], as “injury”, “failure” or “loss” during the first postoperative week.

### Secondary endpoints

The secondary endpoints are:

- Need for new ventilation (invasive or noninvasive) 48 h after the arrest of initial ventilation, during a 21-day postoperative period.
- Renal failure occurring during a 21-day postoperative period according to the RIFLE classification
- Cardiac morbidity: daily serum troponin > 1.5 normal level (specific in each center) within the 48-h postoperative period.
- Cellular and tissue visceral impairment: daily serum D-lactate during the first 8-day postoperative period.
- Mortality at 60 days

This study is randomized in two arms on the day of surgery by drawing lots

- Arm 1: patients with aortic clamping with preconditioning
- Arm 2: patients with aortic clamping without preconditioning

### Eligibility

#### Inclusion criteria

- Patients of at least 18 years
- Signed written informed consent
- Patients with thoracoabdominal aortic aneurysms (TAAA), type 1 to 5 according to Crawford/Safi classification, scheduled for open surgery
- Patients with AAA requiring supra-visceral aortic cross clamping and reconstruction of the visceral arteries
- Patients with TAAA with or without distal femoral perfusion assistance
- Patients with degenerative TAAA or post aortic dissection TAAA
- Patients having read and understood the information letter and signed the Informed Consent Form
- Patients affiliated to, or beneficiary of a social security coverage.

#### Exclusion criteria

- Patients with AAA which require supra-visceral aortic cross clamping but not reconstruction of the visceral arteries (supra-visceral aortic cross clamping for juxta renal aortic aneurysms)
- Patients with TAAA which require a circulatory arrest (aortic aneurysms which affect all the descendant thoracic aorta and the aortic arch)
- Patients requiring emergency surgery
- Patients receiving treatments that may interact with preconditioning such as nicorandil or oral antidiabetics.
- Pregnant women. Women who are not postmenopausal (≥ 12 months of non-therapy-induced amenorrhea) or not surgically sterile must have a negative serum pregnancy test within 1 week prior to randomization.
- Participation in another interventional clinical trial within 28 days prior to randomization and during the study
- Person deprived of liberty by administrative or judicial decision or placed under judicial protection (guardianship or supervision).

### Trial intervention

#### Surgical procedure

Surgical procedures were harmonized between the different centers during a common previous meeting after the protocol edition. The procedure for aortic surgical repair is well established and applied similarly in the different surgical centers. Cerebrospinal fluid drainage will be performed and monitored the operation and for the subsequent 48 to 72 h. Left heart bypass will be used: arterial cannulation will be carried out through the femoral artery and venous cannulation will be placed in the right atrium through the femoral vein. Distal bypass flows will be in range of 1.5 to 2 l/mn. Operation will be conducted with permissive hypothermia. Repair of the aorta will be driven through the sequential aortic clamping technique. When preoperatively identified, intercostal vessels destined for the spinal cord will be reattached to the aortic graft. Otherwise, all patent intercostal arteries from T8 to T12 will be reattached in the main graft. When possible, visceral arteries and the right renal artery will be repaired in a single aortic patch reattached in the main graft. Most of the time, the left renal artery will be repaired separately. Because of the rationale of the study, testing the efficacy of preconditioning for protection against the deleterious effects of ischemia-reperfusion, visceral and renal vessels will not be perfused.

Type IV TAAA will be operated without cerebrospinal fluid drainage and without left heart bypass but according the clamp and go technique.

#### Experimental group

The experimental group consists of patients receiving open TAAA surgery with direct preconditioning by clamping of the supra-visceral aorta.

Randomization allocation will be given to the surgeon during the procedure after intravenous injection of heparin, or after starting femoral assistance. The IPC will be a supra-visceral thoracic aortic cross clamping of 5 min followed by an unclamping period of 5 min. It will be repeated twice before starting the thoracic aortic cross clamping needed to perform surgery. The level of aortic cross clamping for IPC will be that required for aortic repair whose technique has been described elsewhere [35].

Postoperative follow-up will be the same as for standard OSR for TAAA. The follow-up period will cover the duration of hospitalization or will end on the 61st postoperative day in the event of prolongation of hospitalization beyond 60 days.

#### Control group

Patients will receive standard OSR for TAAA without preconditioning.

Postoperative follow-up will be the same as for the experimental group.

### Follow-up

The data collected during the intervention and during the postoperative period are presented in Tables 1 and 2.

**Table 1:** Data collected during surgery

Peroperative data collected	
**Total duration of intervention (mn)**	
**Duration of aortic clamping (mn)**	
**Duration of visceral arteries clamping** **(celiac axis, mesenteric artery, renal arteries)**	
**Duration of distal aortic perfusion (femoral assistance) when used**	
**mL of blood returned by the cell-saver**	
**mL of blood products transfused**	
**Peroperative urine output**	
**Hemodynamic monitoring points (when available): CI / BP / CF** **• before aortic clamping** **• during aortic unclamping** **• after aortic unclamping at 1, 3 and 24 h**	

*CI* Cardiac Index, *BP* Blood Pressure, *CF* Cardiac Frequency

**Table 2:** Data collected during the postoperative period

Postoperative data	
**Duration of invasive or noninvasive ventilation (hours)**	
**Data on ventilatory monitoring for definition of moderate or severe acute respiratory distress syndrome, according to the Berlin definition** [34] **• moderate: (100 mmHg < PaO2/FiO2** **<** **200 mmHg) avec PEEP** **>** **5 cm H2O** **• severe: (PaO2/FiO2** **<** **100 mmHg) avec PEEP** **>** **5 cm H2O**	
**Arterial blood samples: lactates and blood gas at H1, H3 and daily (D1 to D8)**	
**Venous blood samples: serum creatinine at H3 and daily from D1 to D8**	
**Venous blood samples: Troponin on D1, D2**	
**Hourly urine volume output**	
**Duration of hemodialysis, if any**	
**Length of stay in intensive care unit**	
**Length of stay in hospital**	
**Death, if any**	

H: Postoperative hours, D: Postoperative days

### Trial setting

Exclusive involvement of expert centres in aortic surgery with about 150 open aortic surgical procedures per year. The participating University hospitals are Charles Nicolle in Rouen (France), Rangueil in Toulouse (France), La Pitié Salpétrière in Paris, Centre chirurgical Marie Lannelongue in Le Plessis Robinson, La Timone in Marseille (France), Pellegrin in Bordeaux (France), Nouvel Hopital Civil in Strasbourg (France) and Le Bocage in Dijon (France).

### Study schedule

- Enrolment period: 36 months
- Follow-up period: 2 months
- Total duration of the study: 38 months

### Screening visit (day - 60 to day - 5 prior to randomization)

#### Visit 2 baseline (week 0/Day-1)

The baseline visit occur after the patient has signed the informed consent document. All baseline procedures and tests must be completed prior to randomization: review of eligibility criteria, blood chemistry and serum creatinine, health outcomes assessments.

- Day 0: surgery and randomization - Operative period (Table 1)
- Postoperative period (Table 2)

### Statistical methods

The main objective is to study the potential effectiveness of the IPC regarding postoperative pulmonary and renal complications in the two arms during the first 8 days following surgery. As of other complications may occur before the occurrence of a pulmonary or renal complication, a Cox regression model for competing risks will be used to estimate and test the effect of PCI on the occurrence of a postoperative pulmonary complication [36]. The study will be conducted with an alpha risk of .05 for two-sided tests comparing the null hypothesis to the alternative (the risk of a pulmonary or renal complication differs between the two arms). Both arms will be considered to differ significantly if the null hypothesis is rejected.

### Number of patients to be enrolled

The sample size is calculated taking into account an estimated risk of pulmonary and renal complications around 36% following OSR for TAAA in patients operated without PCI [6] and a postoperative mortality around 1% of the patients before the 8th day (observed in the selected centres). With the hypothesis of a corresponding risk in the PCI group around 15%, the randomization of 110 patients, with an allocation ratio of one to one, will detect a Hazard Ratio ≥ 2.75 between both arms with a power of 80% and an alpha risk of 5%.

## Discussion

This project is original since no study has tested PCI as protective against lung and renal damages following OSR for TAAA.

Our hypothesis is that ischemic preconditioning may modulate the inflammatory response associated with visceral ischemia reperfusion caused by the cross clamping of the supra-visceral aorta and thus decrease clinical morbidity, and particularly lung damage. A direct individual benefit is expected with a decrease in mortality and a significant reduction in the total duration of hospitalization and in particular of the days spent in intensive care.

## Data Availability

This study is activated only now. No data are available. Not applicable. The authors have no competing interest to declare. The study received financial support from the French Clinical Research Program (PHRC 2018).
